# Examining inclusivity: the use of AI and diverse populations in health and social care: a systematic review

**DOI:** 10.1186/s12911-025-02884-1

**Published:** 2025-02-05

**Authors:** John Gabriel O. Marko, Ciprian Daniel Neagu, P. B. Anand

**Affiliations:** 1 University of Bradford Facility of Engineering and Digital Technology, Bradford, UK; 2 University of Bradford Faculty of Management Law and Social Sciences, Bradford, UK

**Keywords:** Artificial intelligence, Diverse population, Healthcare, Inclusivity in artificial intelligence, Marginalized population

## Abstract

**Background:**

Artificial intelligence (AI)-based systems are being rapidly integrated into the fields of health and social care. Although such systems can substantially improve the provision of care, diverse and marginalized populations are often incorrectly or insufficiently represented within these systems. This review aims to assess the influence of AI on health and social care among these populations, particularly with regard to issues related to inclusivity and regulatory concerns.

**Methods:**

We followed the Preferred Reporting Items for Systematic Reviews and Meta-Analyses guidelines. Six leading databases were searched, and 129 articles were selected for this review in line with predefined eligibility criteria.

**Results:**

This research revealed disparities in AI outcomes, accessibility, and representation among diverse groups due to biased data sources and a lack of representation in training datasets, which can potentially exacerbate inequalities in care delivery for marginalized communities.

**Conclusion:**

AI development practices, legal frameworks, and policies must be reformulated to ensure that AI is applied in an equitable manner. A holistic approach must be used to address disparities, enforce effective regulations, safeguard privacy, promote inclusion and equity, and emphasize rigorous validation.

**Supplementary Information:**

The online version contains supplementary material available at 10.1186/s12911-025-02884-1.

## Background

### Rationale of the study

Artificial intelligence (AI) is significantly restructuring the healthcare landscape. Healthcare professionals are leveraging AI to enhance diagnostic accuracy, optimize patient-care planning, and improve ongoing monitoring practices [1]. Additionally, AI can be used to navigate through vast medical datasets, revealing hidden patterns and insights that clinicians can use to accelerate decision making and make informed decisions [2]. Furthermore, AI affords advanced problem-solving strategies beyond traditional human capacities, enabling a nuanced approach to medical challenges and supporting cutting-edge and personalized healthcare [3]. However, these advancements are impeded by several challenges. The equitable impact of AI, specifically its effects on diverse and marginalized populations, is attracting considerable attention [4]. These populations already experience systemic healthcare disparities, and improperly designed or intrinsically biased AI systems may perpetuate these disparities [5]. Studies have shown that AI and machine learning (ML) models sometimes fail, specifically for women, individuals from racial minority groups, and individuals with public insurance [6]. Moreover, some models have demonstrated biases, such as recommending disparate treatments based on race and depriving Black patients of crucial care management programs [7, 8]. Despite the recognition of these risks, studies addressing the impacts of AI systems on these populations within the context of health and social care have limitations. Additionally, the current legal and ethical frameworks guiding AI applications often disregard diversity and inclusivity, failing to protect marginalized populations [9, 10].

### Objectives

We systematically reviewed the available literature with the goal of understanding the impacts of the AI systems used in health and social care on diverse and marginalized populations. Marginalized populations were defined in terms of socioeconomic status, race, ethnicity, gender, disability status, and sexual orientation; indigenous individuals, immigrants, and refugees were also included in this category. We evaluated the adequacy of the existing legal and ethical frameworks to the task of ensuring inclusivity and equity in the use of AI in healthcare.

## Methods

To guide the systematic review process from the preliminary search phase to the final screening phase, the Preferred Reporting Items for Systematic Reviews and Meta-Analyses guidelines (PRISMA) [11], were followed in this research. The computer-assisted qualitative data analysis software NVivo-14 [12] (Lumivero) was used to facilitate efficient data management and analysis, and the framework method [13] was employed.

### Eligibility criteria

A comprehensive selection for studies was conducted on the basis of the following eligibility criteria:

1. Studies specifically exploring AI systems’ use and impact within health and social care settings, including diagnostics, treatment, patient monitoring, and administration.
2. Studies on the effects of AI systems on diverse and marginalized populations, within the health and social care.
3. Studies discussing the legal and ethical dimensions of AI in health and social care, especially as they impact diverse and marginalized populations.
4. Original research articles (including qualitative, quantitative, and mixed-methods research), review articles, and case studies published in peer-reviewed journals.
5. Studies published in English only.

### Information sources and search criteria

The sample, phenomenon of interest, design, evaluation, research type (SPIDER) framework [13] was used to formulate eligibility criteria for studies and to develop an effective search string that could ensure that this research employed a comprehensive and rigorous review approach. The SPIDER framework is particularly useful for qualitative and mixed-method research. Table 1. highlights the influence of each component of the SPIDER framework on our search string.

**Table 1:** Components of the SPIDER framework

Component	Framework
Sample	Studies focusing on diverse populations, such as marginalized populations, underrepresented groups, ethnic minority groups, and persons with disabilities. Such terms were included in our search string.
Phenomenon of interest	The application and impact of AI systems in the context of health and social care, which led to the use of search terms such as “Artificial intelligence”, “Machine learning”, “AI systems”, “Health AI”, and “AI in social care”.
Design	Not limited to specific design types.
Evaluation	Addressed by search terms such as “impact”, “effect”, “consequences”, “bias”, and “discrimination”.
Research type	All relevant studies, including quantitative, qualitative, and mixed-methods studies, were included.

The search string developed based on Table 1 was as follows: (“artificial intelligence” OR “machine learning” OR “AI systems” OR “health AI” OR “AI in social care”) AND (“diverse populations” OR “marginalized populations” OR “‘underrepresented groups” OR “ethnic minorities” OR “persons with disabilities”) AND (“impact” OR “effect” OR “consequences” OR “bias” OR “discrimination”).

### Selection and sources of evidence

A methodical search was conducted on June 28, 2023, by using the aforementioned search string in six prominent databases, namely, Google Scholar, Web of Science, Embase, IEEE Xplore, Scopus, and PubMed (MEDLINE). Data, including titles, abstracts, keywords, authors’ names and affiliations, journal names, and publication year, were extracted from the records thus identified. This information was transferred to Sysrev, a web-based platform designed to facilitate data extraction, data curation, and systematic review [14]. Two reviewers subsequently performed a comprehensive assessment of the records thus identified with the goal of determining whether the inclusion criteria were met.

### Risk of bias assessment

The risk of bias in the included studies was systematically assessed by two independent reviewers to minimize individual bias and ensure a comprehensive evaluation. Any discrepancies were resolved through discussion, with a third reviewer consulted if necessary to reach consensus. NVivo 14 was employed to facilitate the qualitative data analysis, as suggested by Jackson and Bazeley [15]. In addition, our analysis adhered to the framework described by Gale et al. [13]. Notably, this method enabled us to make comparisons both within and across cases.

### Data charting and data items

The preliminary search produced extensive data that were efficiently managed using framework matrices with the assistance of NVivo 14 [12]. This tool was used to categorize and examine the data systematically; each row represented an author, while columns indicated different codes or themes that were identified during the literature analysis. This matrix structure provided concise overviews of the approaches to various themes taken by each author.

### Synthesis of the results

**Fig. 1:**
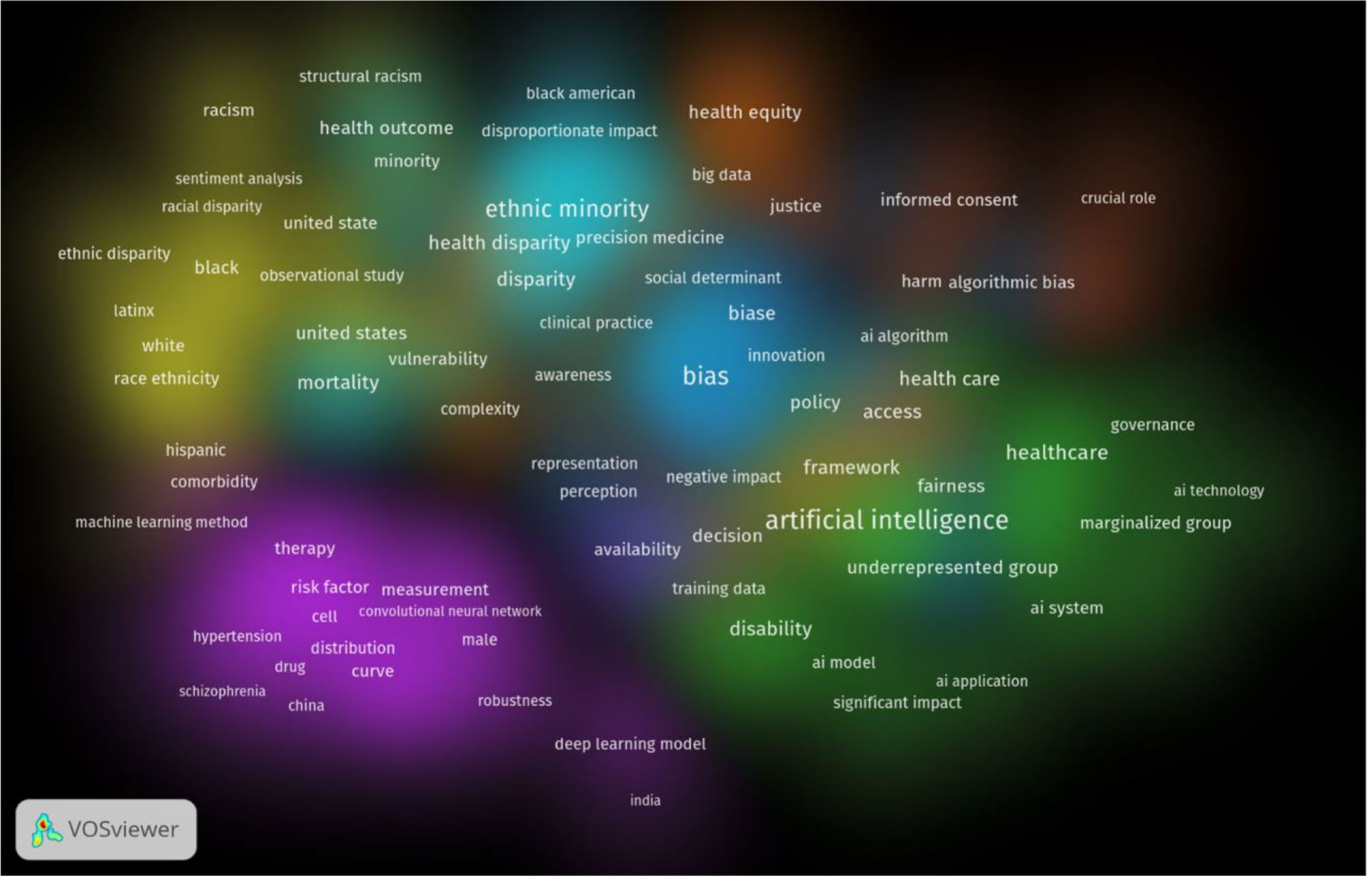
Item density visualization of the co-occurrence analysis of high-frequency keywords

We used a dual approach to analyse the descriptive and conceptual aspects of the studies. First, we examined the foundational data for these studies, including by noting keyword frequencies, as illustrated in Fig. 1. We subsequently used a framework methodology to extract and synthesize emerging themes, note preliminary patterns, and establish a thematic framework on the basis of recurrent issues and concepts. Relevant study segments were assigned to these themes, and the coded data were structured to facilitate comparative analysis. We traced patterns, relationships, and areas of contention across studies pertaining to each theme with the goal of obtaining a comprehensive understanding of the subject.

### Selection of the source of evidence

**Fig. 2:**
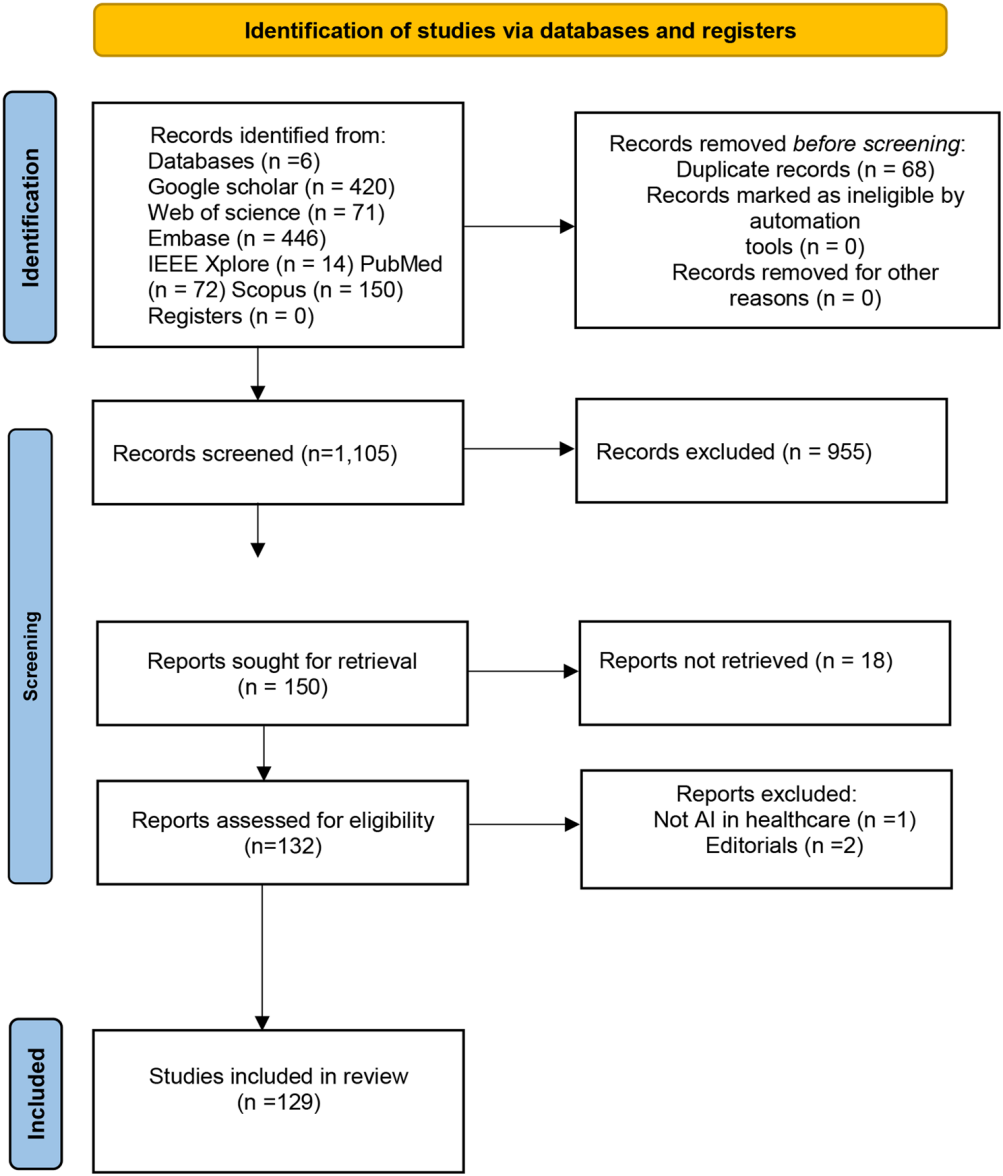
PRISMA flow chart for the stages of the systematic review

We initially identified 1,173 articles. After a preliminary screening, 955 articles were excluded because they did not meet the eligibility criteria, were out of the scope of the study, lacked sufficient methodological rigor, or were published in non-peer-reviewed sources. The remaining 218 articles underwent a thorough evaluation. Among these, 68 were identified as duplicates, 18 could not be retrieved due to subscription barriers, 1 did not address AI in healthcare, and 2 were letters to the editor. Consequently, the final review comprised 129 articles. The design of the search and screening stages is illustrated in Fig. 2.

## Results

### Syntheses of the results

This section presents the synthesis of our findings, which are structured based on the thematic framework described in the Methods section.

#### Bias

Bias refers to the amplification of preexisting disparities, often associated with socioeconomic status, race, ethnicity, religion, gender, disability status, or sexual orientation, which in turn exacerbates inequalities within healthcare systems [16–18]. The integration of AI in healthcare reveals several systemic limitations, notably in the form of racial and ethnic disparities in conditions like cardiovascular disease [19]. Addressing these disparities requires systemic change, focusing on equity rather than solely advancing treatments. Studies show that AI models frequently rely on datasets that fail to reflect the diversity of global patient populations, particularly in areas like medical imaging [20–23]. For example, dermatological AI models may accurately diagnose skin conditions in light-skinned individuals but perform poorly for those with darker skin due to underrepresentation in training data [24]. These disparities extend to underrepresented LGBTQ + communities, a topic that remains under-researched [25, 26].

The COVID-19 pandemic has exposed how bias, discrimination, and racism adversely affect health outcomes [7, 27–32]. The increased adoption of digital healthcare solutions has raised concerns about exacerbating disparities in digital access for disadvantaged populations [33, 34]. Virtual care, for example, may worsen health disparities among underserved communities that lack reliable access to digital technologies [35]. Furthermore, AI systems are susceptible to biases within health information technologies, where the choice of datasets and outcomes can influence unequal care delivery [36, 37]. Such biases can affect the allocation of healthcare resources based on demographic factors or introduce errors into language models used in clinical environments [38]. Racial bias has been observed in algorithms used to assess kidney function, which is critical in diagnosing and managing chronic kidney disease [39]. Similarly, facial recognition algorithms in healthcare may misidentify individuals from minority groups, leading to disparities in care [40, 41].

Previous research on AI in healthcare has primarily used retrospective data, which, while informative, often inherits previous biases and fails to capture real-time clinical nuances [42–44]. Geographic disparities in AI model training further limit the global applicability of these systems and introduce additional biases [42, 45]. Dataset imbalances can compromise the predictive accuracy of AI models, particularly for underrepresented groups [46–48]. Clinical trials, a key part of medical research, also face representation issues. Despite the higher prevalence of conditions like congenital heart disease among Black and Hispanic populations, these groups remain underrepresented in pivotal trials [19, 49]. Inadequate audit mechanisms that fail to account for shifting population risks further heighten the dangers faced by underserved communities [50].

Socioeconomic factors, including education level, residential location, and economic status, significantly impact health outcomes. Women from ethnic minority groups who live in poverty and are subject to gender myths and stereotypes often experience more severe health disparities [51]. For example, the healthcare costs associated with Black patients are often lower than those for white patients, reflecting systemic disparities in care access and barriers such as discrimination and mistrust. Consequently, algorithms that rely on cost as a primary metric may undervalue the healthcare needs of Black individuals [48, 52].

The uncritical use of biased models in clinical decision-making carries significant implications, underscoring the need for caution when applying machine learning in healthcare [53]. While AI holds the potential to extend specialized care to underserved populations, financial barriers could further deepen healthcare access inequalities [54, 55]. Emerging solutions like federated learning offer potential to reduce biases; however, accessibility remains an issue. Smaller medical institutions may lack the resources needed to adopt advanced AI technologies, and the dominance of large corporations in AI could limit its widespread use, thereby perpetuating healthcare inequalities [56].

#### Regulations and policy

The integration of AI into healthcare brings immense opportunities but also significant challenges, making the need for robust regulatory frameworks paramount. While AI can enhance healthcare delivery, it also introduces risks that must be carefully managed. Effective regulations are required to ensure the safety, efficacy, and ethical deployment of AI technologies in healthcare. Guidelines from bodies such as the World Health Organization (WHO) stress the importance of safety and effectiveness, alongside fostering dialogue among key stakeholders—developers, regulators, health workers, and patients [57, 58]. The broader regulatory landscape is evolving, with several countries implementing standards to govern AI’s role in healthcare. However, many regulations remain insufficient in comprehensively addressing the complex issues AI presents. A variety of international standards currently guide the development and deployment of AI in healthcare. For instance, the European Commission’s Trustworthy AI guidelines, the USA’s AI Bill of Rights, and Health Canada’s focus on product safety and data privacy provide frameworks to safeguard AI’s use [59, 60]. The UK’s Medical Device Regulations and the Data Protection Act 2018 also play pivotal roles. Despite these efforts, AI remains prone to bias, and existing frameworks fall short in addressing this bias comprehensively [20, 61, 62]. The need for stronger standards and more detailed benchmarking processes to guide clinical efficacy and cost-effectiveness is evident [63, 64].

One of the primary concerns in AI regulation is ensuring fairness, particularly for minority and underrepresented groups. This is essential for achieving inclusivity in healthcare AI. AI systems must be adapted to respect global cultural norms while actively mitigating biases [65]. For instance, AI’s use in diagnosing rare diseases requires careful consideration, as it may inadvertently lead to discrimination. Strong legal protections, similar to the Genetic Information Nondiscrimination Act of 2008, are needed to safeguard against these risks [66]. Efforts to ensure inclusivity align with the UN’s Sustainable Development Goals, urging healthcare providers to prevent the exclusion of vulnerable populations, particularly women [51].

Another critical challenge lies in regional disparities in AI governance. For example, African countries face significant gaps in AI-related regulations, highlighting the urgent need for digital health strategies and clear frameworks around AI liability [45]. The Global South’s underrepresentation in AI development also raises concerns about the perpetuation of global health disparities and the legacy of colonialism in healthcare access [54, 67, 68]. Such discrepancies illustrate the need for more cohesive global approaches to AI governance. Trust is another key issue in AI’s integration into healthcare, particularly in sensitive areas such as end-of-life care [69]. Concerns about data privacy, patient autonomy, and consent are heightened when AI is involved in critical decision-making processes [58]. Inconsistent interpretations of data protection regulations across different jurisdictions further complicate trust-building efforts [70]. To ensure ethical AI deployment, diverse stakeholder engagement is necessary to safeguard data integrity, patient confidentiality, and fair treatment [71, 72]. Finally, addressing the inherent biases within AI systems remains a significant challenge. AI algorithms must be transparent and accountable, particularly when used in high-stakes contexts like public health and justice [55, 73]. The discrepancies between human and algorithmic decision-making highlight the importance of creating standards to ensure consistency across demographic groups. Detailed performance reports for AI models used in clinical settings are essential to maintain trust and accountability [74]. Additionally, educating healthcare professionals on how to detect and address implicit biases in AI tools can mitigate some of these risks. While AI holds the potential to enhance healthcare, ongoing dialogue among ethicists, developers, and clinicians is critical to developing effective, unbiased AI systems [75, 76].

#### Privacy

Ensuring privacy in AI-driven healthcare applications is a complex challenge that requires careful consideration of inclusivity, equity, and data security. Anonymizing sociodemographic and clinical data is essential for protecting individuals, particularly from minority communities, and enables researchers to monitor health disparities without compromising privacy [77]. While digital healthcare has improved data transfer efficiency, it has also introduced new challenges related to data auditing and security, especially as AI increases the risk of reidentification through both direct and indirect identifiers [55, 56, 78, 79]. AI algorithms can sometimes detect unintended patterns in data, leading to potential privacy violations. This can include inferring sensitive information like ethnicity from medical images or making incorrect diagnoses based on biased data [80]. For example, AI could potentially be used to predict sexual orientation or genetic predispositions, raising ethical concerns about discrimination. These issues highlight the need for robust privacy safeguards and ongoing exploration of ethical principles in AI healthcare applications [50]. Furthermore, AI-based mobile health applications pose risks of data loss, leakage, and manipulation, which threaten individual privacy and security [81]. Protecting patient data and ensuring ownership are vital to preventing the misuse of AI-generated diagnoses or management recommendations that could lead to stigmatization [58, 66]. Parental concerns about the privacy of their children’s health data are particularly relevant in the context of AI in healthcare. Parents may worry about how their child’s data is being used and whether it is shared transparently and consensually [82]. It is crucial that healthcare platforms ensure that sensitive data is handled discreetly and only shared with appropriate professionals and guardians [80, 83]. The rapid increase in data collection during the COVID-19 pandemic has further heightened concerns about the potential for future discrimination against children based on the collected data [58].

#### Inclusion

Inclusion involves ensuring that all individuals, regardless of their unique characteristics, are represented and able to participate fully in any setting [47]. In the context of AI, the lack of diversity in datasets leads to inaccuracies, especially for marginalized groups whose health issues are often overlooked. Therefore, creating balanced datasets and employing diverse metrics are crucial for developing accurate and equitable AI models [47]. AI systems are not inherently neutral, which means that tools should be intentionally designed to prevent bias and promote inclusivity.

Diverse perspectives are essential throughout the AI development process, from conception to evaluation. Incorporating gender, sex, and socioeconomic factors is particularly important in addressing the health and accessibility challenges faced by marginalized populations, including women and individuals with disabilities [84]. This focus on inclusivity enhances the accessibility of AI tools and ensures that they serve a wide range of users [51]. Promoting user-centered design that focuses on accessibility and usability aligns with the broader goal of democratizing AI [55]. Community engagement is crucial for building inclusive AI systems in healthcare. Actively seeking input from marginalized communities throughout the design and implementation of AI systems is essential. This ensures that these tools account for the specific needs and nuances of diverse individuals and communities [85]. For example, involving indigenous communities in the development of AI-powered telehealth solutions can help ensure that these solutions are culturally appropriate and address the unique healthcare needs of these communities. This approach helps AI serve diverse populations more effectively. In addition to community engagement, patient-centric care is another vital aspect of inclusion. By integrating diverse data sources, such as natural language processing (NLP), AI models can capture the lived experiences and narratives of patients, improving personalized care delivery [86, 87]. Finally, creating diverse oversight committees—including experts from various fields and patient representatives—ensures balanced and informed decision-making. Such committees enhance the credibility of AI-driven healthcare research by addressing concerns around inclusivity and helping to ensure that AI systems meet the needs of all populations [88].

#### Equity

AI has a dual role in healthcare equity: it can either be a powerful tool for promoting fairness or a mechanism that exacerbates existing disparities [89, 90]. When designed and applied thoughtfully, AI can fine-tune resource allocation, ensuring that the needs of vulnerable populations are met. However, without intentional efforts to mitigate bias, AI risks perpetuating inequities in healthcare delivery and access [89, 91]. To ensure that AI promotes equitable outcomes, continuous fairness monitoring and inclusive data management are essential. AI models must be built on diverse, representative datasets to prevent biased outcomes that disproportionately affect marginalized groups. For instance, AI could be used to address disparities in preventive screenings by identifying communities with low access to critical services, thus helping to improve healthcare equity. Similarly, ensuring that clinical trials include diverse participant populations can enhance the fairness of AI-driven healthcare systems [92, 93]. Natural Language Processing (NLP) further contributes by integrating diverse data sources, enabling a more comprehensive understanding of patient experiences and improving patient-centered care [89, 94]. A smooth transition from recognizing AI’s potential to the strategies needed for equitable outcomes brings us to the ethical challenges of AI in healthcare. Developing a clear ethical framework is vital, one that prioritizes fairness and equity in algorithmic decision-making [95–97]. A notable concern is the misapplication of algorithms that mistakenly treat race as a biological factor rather than a social construct, leading to biased clinical decisions [98, 99]. To address these issues, experts have proposed a comprehensive blueprint to advance health equity through AI. This approach combines healthcare ethics with technological responsibility, ensuring that AI adheres to the “do no harm” principle while promoting fairness as it continues to shape healthcare [96, 97].

#### Validation

The validation of AI systems in healthcare is essential to ensure their safety, efficacy, and reliability. Although AI research is growing, few applications have undergone the rigorous clinical validation necessary for real-world use. Without proper validation, concerns about reproducibility, generalizability, and algorithmic design persist, limiting trust in AI technologies in clinical settings [42, 56]. Many standards, particularly those involving AI-based medical devices, lack sufficient validation, underscoring the need for real-world evidence to confirm their effectiveness [59, 100]. Machine learning (ML) studies based on electronic health records often lack demographic diversity, which can compromise fairness in AI models. Including diverse training data and ensuring transparency are key to promoting fairness and accuracy. Additionally, improved reporting guidelines can enhance both representation and reproducibility in these studies [73]. Regulatory bodies worldwide have recognized the importance of empirical evidence and foundational methodologies to support the development and validation of AI models, particularly in terms of safety, efficacy, and equity [38, 101, 102]. Comprehensive clinical tests and verifications are crucial for building trust in AI, as these tests determine the precision of AI diagnostics in clinical environments and assess their societal impact [103, 104]. Validating models with diverse patient populations promotes inclusivity and empowers patients by providing clear information about treatment risks and benefits, rather than technical explanations, thus supporting informed decision-making [66, 105]. Validation must also involve analysing independent datasets and tailoring them to clinical outcomes [106]. While AI developers employ various methodologies and datasets, validation remains vital for ensuring effectiveness in different clinical settings, as success in one domain does not guarantee success in another [107, 108]. Moreover, the performance of AI models depends on data quality, variability, and design. Retrospective evaluations have their limitations, making real-time validation crucial for an accurate assessment of AI tools [109–111]. Validation is particularly challenging in resource-limited settings, where data quality and availability may be constrained. Investing in robust data infrastructure can simplify the validation process and improve AI reliability in such environments [112]. Research has shown that validated AI diagnostic tools can serve as supplementary methods to confirm doctors’ recommendations, alleviate patient concerns, and identify discrepancies between AI assessments and clinical evaluations [113]. However, the use of AI without rigorous validation across diverse real-world scenarios can lead to misdiagnoses. AI models require thorough clinical validation, particularly when their diagnoses deviate from established practices [114]. Contextual bias arises when AI models trained on specific subpopulations fail to generalize across broader groups, emphasizing the need for validation in diverse clinical environments [115]. In-depth investigations are necessary to understand the full impact of AI in healthcare, particularly in clinical settings [116, 117]. Furthermore, advancements in health literacy are hindered by measurement challenges and the lack of comprehensive validation across racial and ethnic groups, limiting the development of effective AI-driven solutions [118]. Researchers have proposed the creation of distinct authoritative bodies, such as in the pharmaceutical domain, to rigorously oversee AI validation processes and facilitate AI integration into healthcare [119]. Ethical considerations are critical to the validation process, requiring an understanding of sociocultural factors and sociotechnical systems. Ethical decision-making during model validation must account for trade-offs, and data scientists must possess both ethical and technical skills to navigate these challenges [120].

#### Global impact

The global impact of AI on health and social care is multifaceted, with varying outcomes depending on regional introduction and regulatory approaches [121]. Regional variations in AI adoption highlight significant differences across locations, with developed countries, particularly in North America and Western Europe, being more advanced in integrating AI into healthcare compared to developing nations [122, 123]. These disparities stem from differences in infrastructure, economic resources, and technological readiness, affecting how AI is utilized in healthcare settings. In regions with robust healthcare systems, AI applications are more readily accepted, often leading to improved health outcomes, depending on the nature of the AI-driven intervention [106]. However, geographical disparities in AI efficacy exist across health fields and regions. For example, regions with high AI adoption rates often experience enhanced diagnostic accuracy, better treatment plans, and improved patient outcomes [107]. Conversely, in areas with insufficient resources or underdeveloped healthcare infrastructures, the impact of AI is less pronounced, potentially leading to disparate health outcomes [124, 125]. The regulatory landscape for AI in healthcare also varies significantly across countries. Ethical, legal, and privacy concerns related to AI use differ depending on regional regulatory frameworks. Countries with well-established regulations are better equipped to address issues such as data protection, algorithmic bias, and patient privacy [126]. Additionally, ethical considerations regarding the global use of AI in health and social care are influenced by regional differences in cultural, linguistic, and socioeconomic diversity, which require tailored approaches to AI implementation [127]. Geographical and socioeconomic factors play a crucial role in determining the availability and accessibility of AI-powered healthcare services in different regions. Areas with wide economic disparities face challenges in ensuring equitable access to AI technologies, potentially exacerbating existing health inequalities if these challenges are not addressed [128, 129].

#### Public perceptions

Recent research on public perceptions and trust in AI-driven health interventions has revealed evolving attitudes, which are crucial for assessing AI’s overall impact on healthcare and social care [106, 130]. A key focus of these studies has been the growing public awareness and education surrounding AI in healthcare. As individuals gain more knowledge about AI’s potential benefits and limitations, their attitudes begin to shift [107, 131]. Educational programs play a vital role in correcting misconceptions and building trust, especially among groups with varying levels of familiarity with AI technologies [132]. Beyond increasing awareness, building trust is essential for the successful integration of AI into healthcare. Trust-building efforts by healthcare institutions and AI developers are critical to securing public acceptance. Open discussions about AI’s use in healthcare, particularly those that emphasize data privacy, bias reduction, and fairness, can significantly enhance public confidence in AI technologies. Incorporating diverse user feedback during the development process ensures that AI systems are reliable and reflect the values of different social groups [133, 134]. Additionally, cultural sensitivity in AI design and deployment has been shown to improve public trust. AI technologies that respect and integrate cultural norms and values are more likely to be seen as thoughtful and respectful, increasing trust across diverse populations [131, 135–138]. Ethical considerations and accountability measures also play a key role in shaping public perceptions. When people believe that AI systems adhere to ethical principles and are accountable for their decisions, their trust in the technology strengthens.

Public trust is further enhanced when AI technologies demonstrate awareness and respect for cultural differences within healthcare practices. Culturally sensitive AI applications are perceived as more considerate, which fosters trust among diverse groups [69,139]. Bias in AI algorithms is another major factor influencing public perception. Studies show that people, particularly those from marginalized communities, are more likely to trust AI systems that actively mitigate biases. Promoting fairness and equality in AI applications has a positive impact on public trust, especially among diverse populations [140, 141].

The intersectionality of trust dynamics has emerged as a key theme in recent studies. Trust in AI-driven healthcare interventions is influenced by multiple factors, such as race, gender, socioeconomic status, and culture. Understanding these intersecting dynamics is essential for tailoring communication strategies and trust-building initiatives to specific demographic groups [142, 143]. Public attitudes towards AI reflect a mix of optimism and apprehension. On the positive side, many people appreciate AI’s potential to improve health, advance scientific discovery, and enhance efficiency. However, concerns persist around the impact of AI on decision-making, privacy, and the need for regulation. Ethical issues, such as bias and discrimination, also play a significant role in shaping public perceptions of AI. Addressing these concerns is critical to responsible AI development and governance in healthcare.

## Discussion

### Discussion of the main results

This systematic review has illuminated the complex landscape of AI integration in healthcare, revealing a terrain marked by both transformative potential and significant challenges. While AI offers promising advancements in diagnostics, treatment, and patient care, it also raises critical concerns about bias, regulation, privacy, and inclusion, particularly for marginalized populations. To systematically analyse these findings, Table 2 provides a comprehensive framework categorizing the key parameters and considerations across eight critical domains affecting AI implementation in healthcare settings. The table reveals the interconnected nature of challenges facing AI adoption in healthcare, from bias and regulatory concerns to privacy and public perception. Each category represents a crucial aspect of healthcare AI implementation that must be carefully considered to ensure equitable and effective deployment.

**Table 2:** Comparison based on different parameters

Category and parameter	Key findings and considerations
**Bias**	
Race and ethnicity bias	Bias in AI-based medical imaging for light-skinned individuals.
Gender bias	Health disparities for women in ethnic minority groups.
Geographical disparities	Amplification of bias in retrospective studies.
Clinical trial bias	Minimal representation of certain populations, which raises efficacy concerns.
Socioeconomic bias	The undervaluation of healthcare costs for certain demographic groups, which affects algorithms.
Algorithmic bias in various applications	Biases in algorithms used to determine kidney function and perform facial recognition.
Federated learning as a solution	Potential accessibility issues for small institutions and corporate dominance.
**Regulations and policy**	
International norms	Recommendations from the WHO, the USA’s AI Bill of Rights, and the European Commission.
Fairness and health inequities	Need for strong regulatory standards and guidelines to address potential health inequities.
Dedication to diversity	Legislative protections for the use of AI to address rare diseases in line with the UN’s Sustainable Development Goals.
Uniform legal frameworks	A lack of such frameworks, which entails compliance challenges, thus highlighting the necessity of state supervision.
**Privacy**	
Challenges pertaining to data transfer	The simplification of data transfer through digitalization, which nevertheless introduces challenges related to security and auditing.
Ethical principles	Inadequate exploration of the influence of ethical principles on AI models.
**Need for AI regulations**	The necessity of AI regulation in healthcare, especially with regard to unintended causal patterns.
**Inclusion**	
Balanced datasets	Essential for model quality and the avoidance of errors.
Community engagement	Essential for avoiding biases; inclusivity is a moral and strategic imperative.
Patient-centric AI	The need for AI to incorporate gender, sex, and socioeconomic factors comprehensively.
**Equity**	
Dual effects of AI	The fact that AI may either promote or impede health equity.
Addressingvulnerable populations	An emphasis on the needs of vulnerable populations through equitable data management and testing methodologies.
NLP in patient-centric care	Identification of NLP as a powerful tool for patient-centric care, which can promote equity.
**Validation**	
Challenges pertaining to clinical validation	Challenges that highlight the need for real-world evidence and comprehensive testing methodologies.
Importance of empirical evidence	A regulatory emphasis on empirical evidence to support the safety, efficacy, and equity of the use of AI in healthcare.
Nuanced model performance	The necessity of validation in diverse domains.
**Global impact**	
Regional disparities in adoption	The fact that developed countries exhibit advanced integration, thus leading to variations in healthcare outcomes.
Variations in outcomes and efficacy	Geographical disparities, which result in varying outcomes and context-dependent effectiveness.
Ethical considerations	Essential for inclusive AI deployment.
**Public perceptions**	
Awareness and education	The positive influence of increased awareness on perceptions, especially among individuals with diverse backgrounds.
Trust-building measures	Transparent communication and community engagement, which contribute to the establishment of trust.
Cultural sensitivity in AI design	A positive influence on public trust by respecting diverse norms and values.
Community engagement	Community engagement in decision-making processes, which establishes trust.
Ethical considerations and accountability	Public trust, which is influenced by ethical frameworks and clear accountability measures in the context of AI applications.
Addressing bias and fairness	Efforts to enhance fairness and equity, which resonate positively with diverse populations.
Intersectionality in trust dynamics	The recognition of intersectionality in trust dynamics, including the fact that trust is influenced by various factors such as race, gender, and socioeconomic status.

Our analysis identified pervasive biases in AI models, notably related to race, gender, and socioeconomic status, similar findings have been reported in recent studies, such as [144, 145], which corroborates our observations and highlights the urgency of addressing these biases. These biases are deeply rooted, stemming from unrepresentative datasets, algorithmic design, and societal biases embedded in the data itself. Specific instances of bias were evident in the literature, such as dermatological AI systems that may misdiagnose skin conditions in individuals with darker skin tones due to underrepresentation in training datasets. Similarly, algorithms prioritizing cost-effectiveness over individual needs could inadvertently disadvantage patients from marginalized communities who often require more complex care [20–26]. These biases can have far-reaching consequences, impacting diagnostic accuracy, treatment decisions, and resource allocation, ultimately affecting patient outcomes and exacerbating health disparities.

The current regulatory frameworks for AI in healthcare are struggling to keep pace with its rapid evolution and unique challenges [146]. Existing regulations often lack specificity and does not sufficiently account for the distinct attributes of AI, such as its capability to create synthetic imaging for medical diagnostics, augmenting traditional imaging techniques and potentially leading to earlier and more accurate diagnoses. However, regulations need to address the validation and safety of such AI-generated images. AI algorithms can continuously learn and refine their predictions of patient outcomes based on real-time data analysis. This evolving nature of AI necessitates adaptive regulatory oversight to ensure ongoing accuracy and reliability. This lack of regulatory clarity hinders effective oversight and poses risks to patient safety [58–62]. A more dynamic and adaptive regulatory approach is urgently needed, one that can evolve alongside AI technology while mandating transparency, explainability, and regular audits for bias and discrimination. This approach should consider the entire lifecycle of AI in healthcare, from development and validation to deployment and ongoing monitoring, ensuring that AI technologies are used safely, ethically, and effectively for the benefit of all patients.

Privacy concerns, particularly for minority communities, emerged as a critical area of concern. Unintentional release or breaches of sensitive data, such as ethnicity or social status, can exacerbate existing disparities and fuel further bias in AI systems [55, 56, 78–80]. Robust privacy safeguards, including data minimization techniques and de-identification methods, are essential to protect patient privacy and prevent violations that disproportionately affect vulnerable populations.

The review underscored the dual role AI can play in either exacerbating or mitigating health inequities. To ensure AI serves as a tool for equity, proactive measures are necessary. These include developing and implementing bias mitigation algorithms, promoting the use of explainable AI (XAI) to foster transparency, ensuring diversity in development teams to incorporate a wider range of perspectives, and conducting community-based testing to evaluate AI systems in real-world settings and identify potential disparities.

Addressing these challenges requires a fundamental shift in how we integrate AI into healthcare systems. This necessitates international collaboration to establish global standards and practices that promote inclusivity, transparency, and fairness in AI development and deployment. Robust ethical frameworks are needed to guide responsible AI use, ensuring patient autonomy, data privacy, and equitable access to care. Continuous monitoring and evaluation mechanisms are crucial to identify and address emerging biases and ethical concerns in evolving AI systems.

Beyond the need for further research, this review points to systemic issues in AI integration into healthcare. AI has often emphasized pre-existing disparities, particularly around areas like racism, sexism, and socio-economic biases. These biases are manifestations of more far-reaching societally rooted problems that AI has unwittingly reflected and amplified. Another major challenge is that AI further expands existing disparities in access to digital healthcare, particularly for marginalized communities who may lack digital literacy or access to adequate infrastructure [16–18]. This digital divide can deepen health inequities and must be addressed through targeted investments and inclusive design.

The ethical issues identified in this review are dire and multifaceted. Algorithmic bias in healthcare is more than a technical flaw; it is an ethical failure with real consequences for health outcomes, often disproportionately impacting minorities [39]. Biased datasets and privacy concerns further compound these issues. Existing regimes governing AI in health need urgent rectification, with a necessity for more robust, enforceable global standards.

### Limitations

While this review thoroughly explores the integration of AI into healthcare, several limitations must be noted regarding the interpretation of its findings; these limitations also highlight directions for future research. The search terms used, while broad, may not have captured the full spectrum of relevant literature. Focusing on descriptors like “impact” and “discrimination” might have missed studies that used alternative terminology to address similar concepts (e.g., “fairness,” “equity,” “justice”). Future reviews could incorporate a wider range of search terms to ensure a more comprehensive and nuanced understanding of the ethical implications of AI in healthcare. Additionally, the overlap in meaning among terms like “diverse populations” and “underrepresented groups” might have led to the inclusion of some repetitive articles, potentially skewing the analysis. Future reviews could employ more precise definitions and inclusion/exclusion criteria to mitigate this issue. This review focused on English-language publications, potentially excluding valuable research published in other languages. This language bias could limit the generalizability and comprehensiveness of the findings. Future research should strive to include non-English publications, perhaps through collaboration with international researchers or by utilizing translation services. While Google Scholar was included as a source, the extraction process was not exhaustive due to limitations in the API and the sheer volume of results. Relying on the first 420 articles from a potential pool of over 16,000 might have introduced selection bias. Future research could employ more comprehensive search strategies within Google Scholar or consider manual screening to ensure a more representative sample of relevant literature. The review reveals significant disparities in AI adoption and implementation, in which context developed countries have outpaced developing regions. This geographical imbalance limits the generalizability of the findings of this review, which may overlook the unique challenges associated with low-resource settings, particularly given the varying levels of technological infrastructure across regions. Furthermore, the methodological rigor of the included studies was inconsistent. Some studies lacked robust validation, transparent reporting, and detailed methodological descriptions, thus impacting the overall reliability and reproducibility of their findings. The predominance of cross-sectional studies, although they provided snapshots of the impact of AI in this context, fails to capture long-term outcomes and the evolving nature of AI technologies in healthcare. Although ethical considerations were addressed, a deeper exploration of the principles guiding the development and deployment of AI is needed. Issues pertaining to privacy, patient autonomy, and commercial interests require a thorough investigation that can establish robust ethical frameworks for responsible AI use. Translating research into practice remains challenging, and many studies have highlighted the difficulties associated with scaling and ensuring reproducibility in clinical settings. This limitation highlights the need for practical, adaptable AI solutions that can be seamlessly integrated into existing healthcare systems. While public perceptions were mentioned, a more nuanced analysis of the barriers to acceptance and the roles of education and trust-building in this context is warranted. Understanding diverse perspectives on AI and the factors that influence the acceptance of this technology is crucial with respect to efforts to promote public trust and engagement. Finally, the lack of transparency exhibited by some studies in terms of methodologies and potential conflicts of interest raises concerns regarding the credibility and impartiality of their findings. Clear reporting of funding sources, biases, and methodological details is essential for the establishment of trust in research on AI and its applications.

## Conclusions

This review highlights a crucial reality, as we integrate AI into the intricate fabric of healthcare, we must proceed with caution, guided by ethical considerations and a steadfast commitment to patient well-being. Privacy, equity, and inclusion are not mere buzzwords; they are essential principles that must shape the development and application of AI. AI cannot function in isolation, oblivious to the diverse needs of society; it must be inclusive and representative of all, or risk exacerbating the very healthcare disparities it aims to eliminate. We stand on the brink of a healthcare revolution, where AI’s transformative potential can only be fully realized when it is deeply rooted in ethics and human values. From safeguarding privacy to combating algorithmic bias, it is evident that a collaborative effort is required: ethicists, clinicians, policymakers, and technologists must unite to navigate these complex and uncharted waters.

The path ahead is fraught with challenges. Scholars must develop innovative methods that balance privacy with fairness, while regulatory bodies worldwide must keep pace with the rapid advancements of AI. The true promise of AI lies in its ability to be universally accessible, ensuring that its benefits reach everyone, regardless of economic status. As we advance, we must not shy away from the difficult questions. We need to engage more deeply with the ethical and legal complexities that AI introduces, ensuring that its development and deployment remain transparent and accountable. The stakes are high, but the potential rewards a future where healthcare is equitable, accessible, and powered by intelligent technology are extraordinary.

## Electronic supplementary material

Below is the link to the electronic supplementary material.

Supplementary Material 1

Supplementary Material 2

## Data Availability

The datasets used and analysed during the current study are available in the Sysrev repository. https://www.sysrev.com/register/8Qk17RzkH8NgYayFnooxC1yBNPdK0JZR.

## Abbreviations

AI: Artificial intelligence
ML: Machine learning
PRISMA: Preferred Reporting Items for Systematic Reviews and Meta-Analyses
SPIDER: Sample, phenomenon of interest, design, evaluation, research type
RDs: Rare diseases
NLP: Natural language processing
