# Supplementary material for: Examining inclusivity: the use of AI and diverse populations in health and social care: a systematic review
Source: BMC Med Inform Decis Mak. 2025 Feb 5;25:57. doi: 10.1186/s12911-025-02884-1 (PMC11796235; doi:10.1186/s12911-025-02884-1)
Supplement: Supplementary file 1 — Supplementary Material 1 [file 12911_2025_2884_MOESM1_ESM.docx]

Examining Inclusivity, The use of AI and Diverse Populations in Health and Social Care A Systematic Review

Codes

| Name | Description |
| --- | --- |
| How are these impacts currently addressed in legal and ethical frameworks | Question one |
| Accountability Mechanisms | Systems, procedures, or methods put in place to ensure entities, such as individuals, organizations, or governments, are held responsible for their actions, decisions, and policies. In various contexts. |
| Enforcement Issue | Challenges or problems encountered in ensuring compliance with laws, regulations, policies, or rules. It can pertain to the inability or failure of responsible entities or bodies to implement or enforce established norms or standards effectively. |
| Equity considerations | The deliberate reflection and integration of fairness, justice, and impartiality within policies, practices, and decisions. |
| Ethical guidelines | Represents existing ethical guidelines or considerations relevant to the use of AI in health and social care |
| Inclusion in Decision-making | The practice of involving a diverse group of individuals, who are representative of various backgrounds, perspectives, and abilities, in the process of making decisions |
| Legal Measures | The collection of laws, regulations, guidelines, and ethical standards that are formulated and implemented to govern the development, deployment, and use of AI technologies. |
| Policy Gaps | The lack or insufficiency of guidelines, regulations, or laws governing the development, deployment, and use of AI technologies, leading to a range of ethical, social, and technical challenges |
| Privacy and Consent | The protection of individual’s personal information and the requirement of obtaining explicit permission to collect, store, process, or share such information |
| Regulations and policy | Represents existing laws and regulations relevant to the use of AI in health and social care |
| Validation | The process of evaluating, verifying, or confirming the accuracy, reliability, and effectiveness of a system, process, design, or claim. |
| Legal Challenges | Issues arising from the development, deployment, and use of artificial intelligence technologies. |
| Transparency Issues | The challenge of making AI systems, decisions, and processes clear, understandable, and explainable to users, regulators, and other stakeholders. |
| Opaque Black Box | Systems where the internal workings and decision-making processes are not transparent or understandable to users, developers, or other stakeholders. |
| Reduce Autonomy | Implies imposing restrictions on the extent to which systems can operate, make decisions, or perform actions independently, without human intervention or oversight. |
| What are the potential impacts of AI systems on diverse and marginalized populations in health and social care | Question tow |
| Bias | The presence of systematic and unfair discrimination in the output of algorithms |
| Improved accessibility | The availability, usability, and inclusivity of AI technologies for people with a broad range of abilities, needs, and backgrounds. |
| Improved quality of care | The enhancement of health service delivery to ensure better patient outcomes, safety, and satisfaction |
| Artificial Intelligence | Artificial Intelligence (AI) in health and social care refers to the use of machine learning algorithms and software, or "AI systems", to mimic human cognition in the analysis, interpretation, and comprehension of complex medical and healthcare data |
| Patient Monitoring and Care | The use of AI wearable to monitor patients' vital signs in real-time and alert healthcare professionals if there are concerning changes. AI chatbots that provide mental health support, respond to patient queries, or remind patients to take their medication. |
| Precision Medicine | AI can be used to personalize treatment plans based on an individual's genetics, lifestyle, and environment |
| Predictive Analytics | By analysing large amounts of data, AI can help predict disease outbreaks or patient deterioration, allowing for preventative measures or early interventions. |
| Social Care | the use of AI in assistive technology to support individuals with disabilities or elderly individuals. For instance, AI-powered devices can provide reminders, assist with mobility, or monitor for emergencies |
| Challenges | Technical, legal, ethical, and organizational obstacles. |
| Discrimination | Discrimination can take a variety of forms, often arising from biases embedded in the data used to train AI models, the design of the algorithms themselves, or their deployment. These biases can lead to unequal treatment or outcomes for certain groups of people. |
| Algorithmic Bias | the algorithms themselves can introduce bias, especially if they are designed or optimized in ways that inadvertently Favor certain groups over others. For example, if an AI system in social care is optimized to reduce overall costs, it might disproportionately recommend services or treatments that are less effective for marginalized populations. |
| Bias in Training Data | If the data used to train an AI system does not adequately represent certain population groups, the system may not perform as well for these groups. For instance, many AI models in healthcare have been trained predominantly on data from individuals of certain ethnicities, ages, or genders, which can lead to less accurate predictions or treatments for underrepresented groups |
| Diverse populations | Diverse populations refer to different groups of people that are identified based on various factors, such as race, ethnicity, age, gender, socioeconomic status, disability status, geographical location, sexual orientation, etc |
| Fairness | Fairness refers to the equitable treatment and outcomes for all individuals or groups, irrespective of their specific characteristics such as race, ethnicity, gender, socioeconomic status, age, etc. |
| Health Disparities | The differences in health status, health outcomes, and the quality and accessibility of healthcare among different population groups |
| Access to AI-Powered Services | AI-powered health services, such as telehealth and personalized medicine, require certain infrastructural prerequisites like high-speed internet and advanced healthcare facilities. Marginalized populations might not have access to such resources due to economic, geographical, or social barriers, resulting in inequitable access to these potentially life-saving technologies |
| AI Literacy | The ability to understand and interact effectively with AI systems is not uniformly distributed across the population. Lower levels of digital literacy or AI literacy among certain groups can prevent them from fully benefiting from AI applications in healthcare. |
| Data Bias | AI systems are usually trained on large datasets. If these datasets do not represent certain populations (often the marginalized ones), the AI models can perform poorly when applied to these groups. This could lead to misdiagnosis or ineffective treatment recommendations. For example, skin cancer detection AI systems trained mostly on lighter skin types might underperform when used on darker skin types, thus exacerbating health disparities |
| Health outcomes | The possible results that may come from specific health care services or interventions, including changes to health status, patient satisfaction, health behaviours, knowledge, and health care costs. |
| Marginalized populations | Marginalized populations refer to groups that are underrepresented in data sets, have less access to AI-powered services, or are more susceptible to the negative consequences of these systems. This could be categories such as gender, race, ethnicity, socioeconomic status, children, Disability status |
| Reliability | The ability of a model or system to consistently perform a task and produce stable, dependable, and accurate results under different circumstances, input data, or operational environments |
| Social determinants | The conditions in the environments where people are born, live, learn, work, play, and age that affect a wide range of health, functioning, and quality-of-life outcomes and risks. |
| Socioeconomic factors | The social and economic experiences and realities that influence an individual's perspectives, behaviours, and lifestyles |
